# Arthroscopic Double EndoButton Adjustable‐Loop Plate Fixation for Acute Acromioclavicular Joint Dislocation

**DOI:** 10.1002/atn2.70366

**Published:** 2026-11-15

**Authors:** Shenbo Huang, Zhenxin Zheng, Chao Qian, Hang Yuan, Huricha Bao

**Affiliations:** ^1^ Orthopedics Department Quzhou Affiliated Hospital of Wenzhou Medical University, Quzhou People's Hospital Quzhou China

## Abstract

Acute acromioclavicular joint dislocation is a shoulder injury. Surgical intervention is typically recommended for Rockwood type IV to VI injuries with significant displacement. Both traditional open surgery and existing arthroscopic techniques have limitations in terms of trauma, stability, and procedural complexity. This article introduces an arthroscopic fixation technique using a double EndoButton adjustable‐loop plate. It aims to achieve anatomical reduction and sustained compressive fixation of the acromioclavicular joint through a dual‐EndoButton structure and an adjustable‐loop design. This method is relatively straightforward to perform, minimally invasive, and biomechanically sound, offering a reliable new option for the minimally invasive treatment of acute acromioclavicular joint dislocations. Preliminary clinical practice has shown favorable reduction outcomes, stable fixation performance, and potential for early rehabilitation.

## Graphical abstract

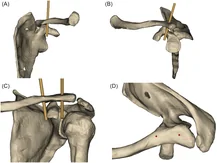

**VIDEO 1:** Undergoing endoscopic surgery with the adjustable double EndoButton plate. Video content can be viewed at https://doi.org/10.1002/atn2.70366.

The acromioclavicular joint, a key structure connecting the distal clavicle to the acromion, plays a crucial role in maintaining shoulder stability and coordinating upper limb movement.^1^ Acromioclavicular joint dislocations account for approximately 9% to 12% of all perishoulder injuries, with an incidence as high as 40% in athletic populations, predominantly affecting young adults engaged in high‐intensity activities.^2^ Currently, clinical diagnosis primarily relies on imaging examinations, and the Rockwood classification system is widely used to assess the severity of injury.^3^ Generally, Rockwood type I and II injuries, characterized by minimal displacement, are predominantly managed conservatively. In contrast, surgical intervention is typically recommended for Rockwood type IV and VI injuries with significant displacement.^4^

Existing surgical techniques include hook plate fixation, suture anchor techniques, TightRope (Arthrex, Naples, FL)/EndoButton suspension systems, high‐strength suture cerclage, and tendon grafts.^5^, ^6^, ^7^, ^8^ Despite the variety of available procedures, controversy persists regarding the optimal surgical plan, particularly concerning how to achieve stable anatomical reduction, maintain long‐term stability, and maximally preserve shoulder function. Traditional open surgery is associated with greater trauma, whereas existing arthroscopic techniques also face challenges such as procedural complexity and implant adaptability.^9^, ^10^

To address these issues, we describe an arthroscopic double EndoButton adjustable‐loop plate fixation technique for the treatment of acute acromioclavicular joint dislocations. This technique, using a double EndoButton construct and an adjustable‐loop design, aims to achieve a more physiological mechanical reconstruction, provides sustained and stable compression fixation, and offers advantages such as relative procedural simplicity and reduced trauma. This article systematically describes the operational steps, preliminary clinical application outcomes, and potential clinical value of this technique, with the aim of providing a reliable option for the minimally invasive treatment of acromioclavicular joint dislocations.

## SURGICAL TECHNIQUE

### Patient Evaluation and Indications

Indications for this technique include patients with acute closed acromioclavicular joint dislocation (injury duration ≤3 weeks) and radiographic confirmation of Rockwood types IV to VI. Contraindications include combined injuries such as concomitant acromial fracture, clavicular fracture, coracoid process fracture, severe soft tissue injury, or neurovascular injury.

### Preparation Before Surgery

Preoperative diagnosis is confirmed via imaging. Based on computed tomography data, 3‐dimensional reconstruction of the acromioclavicular joint is performed using Mimics software to plan the positions of bilateral bone tunnels in the coracoid process (Figure 1). The distances from the bone tunnels to the distal clavicle and the linear distance from the coracoid tip to the clavicular surface are measured. Surgery is performed under general anesthesia with the patient routinely placed in the lateral decubitus position. After sterile preparation and draping, the clavicle and coracoid process are marked, and the bone tunnel entry points are delineated according to the preoperative measurements (Figure 2).

**FIGURE 1:**
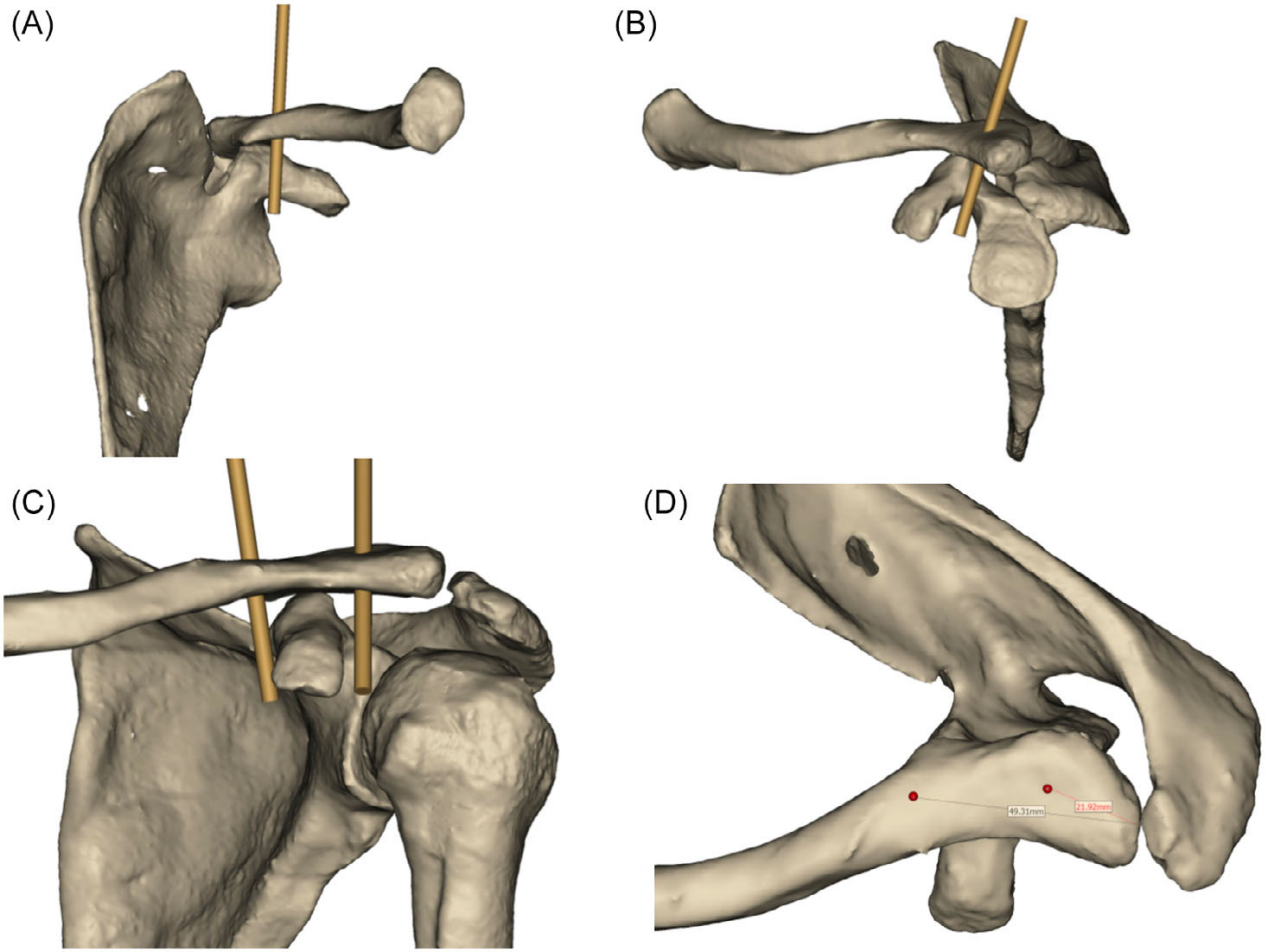
Three‐dimensional reconstruction of the acromioclavicular joint using Mimics software. (A,B) Planning the positions of bilateral bone tunnels in the coracoid process and the drilling directions. (C) Anteroposterior view. (D) Measuring the distance from the bone tunnel to the distal clavicle.

**FIGURE 2:**
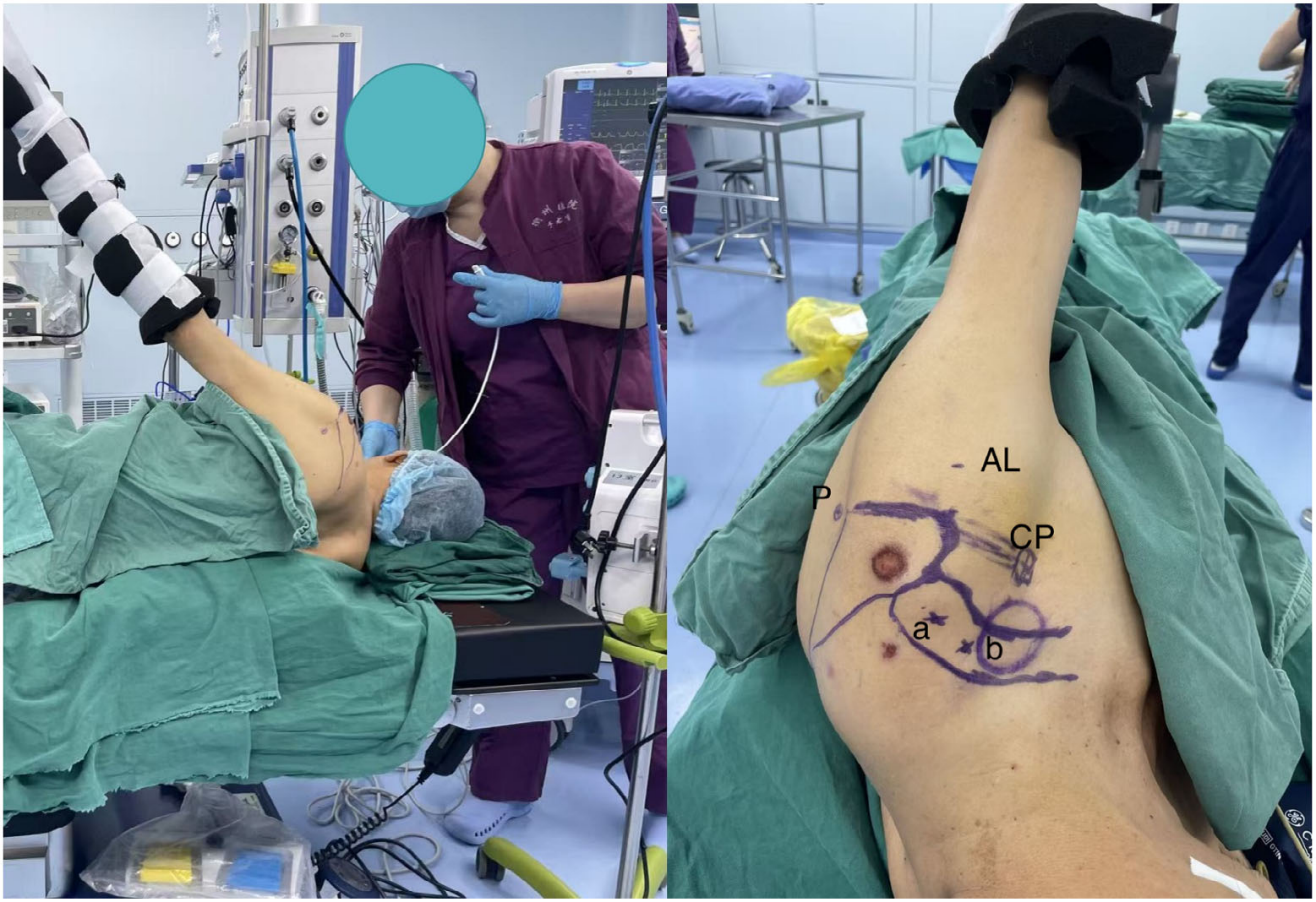
The patient is placed in the lateral decubitus position for surgery. The clavicle and coracoid process are marked, and the bone tunnel entry points are determined based on preoperative measurement data. (a and b, the medial and lateral planned bone tunnel entry points; AL, acromion lateral border; CP, coracoid process; P, posterior.)

### Arthroscopic Exploration

Prior to arthroscopy, 2 long needles are used to localize the anterior and posterior borders of the clavicle. A 2.0‐mm Kirschner wire is then inserted along the markings to establish the bone tunnel. Intraoperatively, care is taken to ensure that the drill bit penetrates only the double cortex of the clavicle to avoid injury to the subclavian vessels and nerves (Figure 3). Subsequently, a 4.5‐mm EndoButton drill is introduced along the Kirschner wire channel to enlarge the bone tunnel. A 0.5‐cm posterior portal incision is made 2 cm inferior and 1.5 cm medial to the posterolateral corner of the acromion. The arthroscope is inserted to explore the glenohumeral joint and assess for concomitant pathologies such as rotator cuff tear, labral injury, or glenoid fracture. An anteroinferior portal is established between the coracoid process and the anterolateral acromion, and a shaver and radiofrequency device are used to perform synovectomy at the base of the coracoid process. An anterolateral portal is then created approximately 2 cm lateral to the anterolateral corner of the acromion to serve as the viewing channel for subsequent procedures.

**FIGURE 3:**
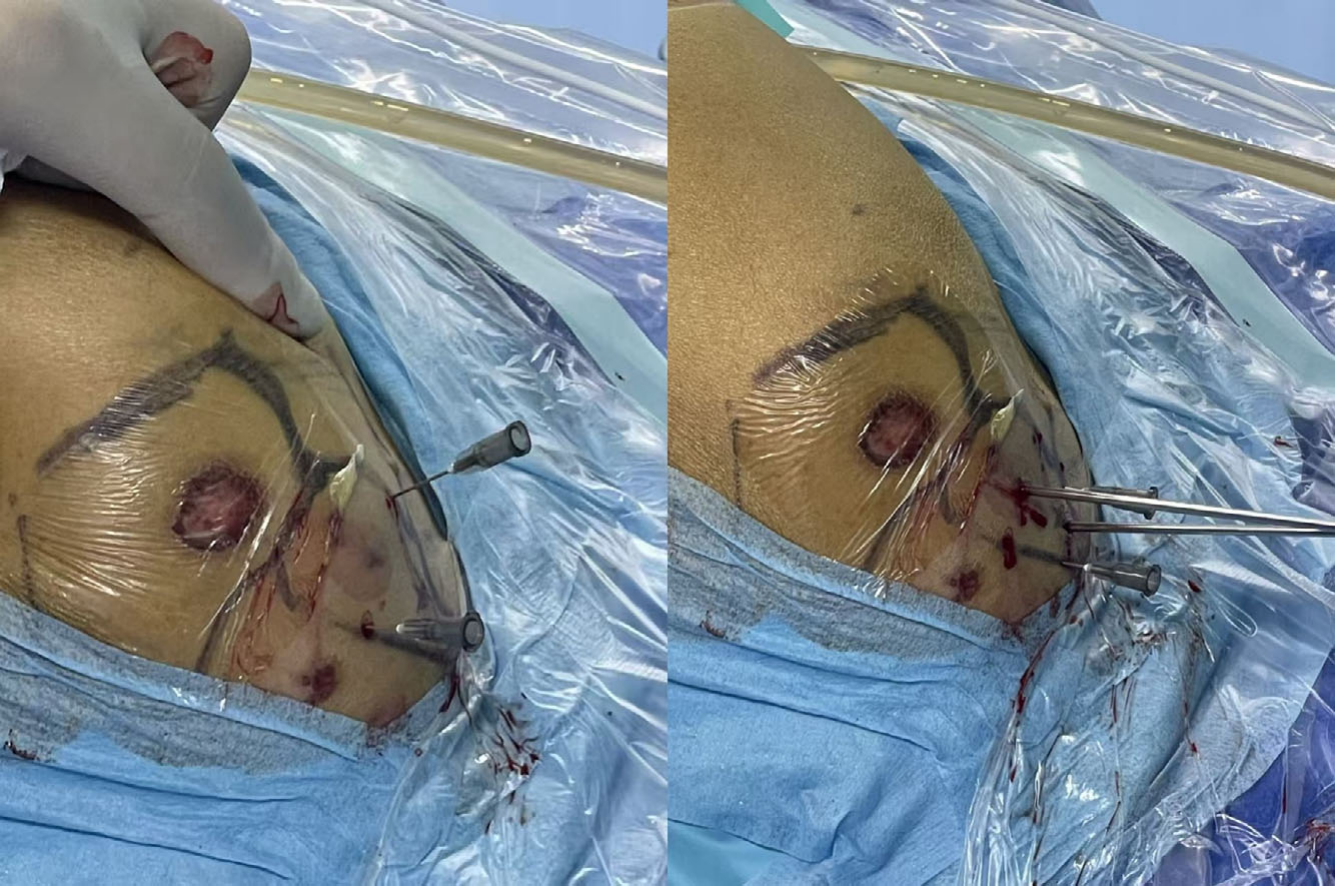
Two long needles are used to locate the anterior and posterior borders of the clavicle. A 2.0‐mm Kirschner wire is then used to create the bone tunnel according to the markings.

### Passage of Guide Lines Through Bone Tunnel

A knot pusher is used to pass polydioxanone sutures through the medial and lateral 4.5‐mm bone tunnels to the inferior surface of the coracoid process. Under direct arthroscopic visualization, both sutures are grasped into the same channel using a suture retriever. A lasso configuration is employed to thread the double EndoButton adjustable‐loop plate (Beijing Tianxing Medical, Beijing; P2506447) into a single unit, which is then pulled out through the respective bone tunnels (Figure 4).

**FIGURE 4:**
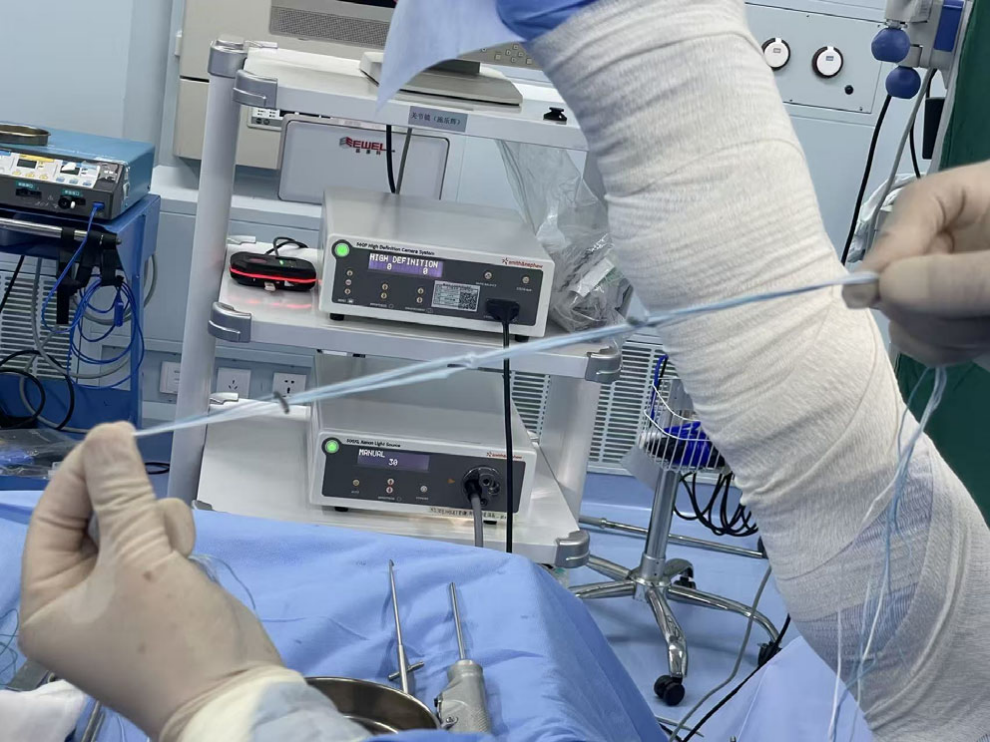
The 2 EndoButton adjustable‐loop plates are integrated using a lasso configuration.

### Reduction and Final Fixation

Manual reduction is performed by depressing the clavicle while simultaneously lifting the elbow. After satisfactory reduction is achieved, the adjustable‐loop plate is tensioned and secured to maintain anatomical alignment. During the tightening process, the lateral adjustment loop is tightened first to ensure its fixation on the superior surface of the clavicle, followed by tightening of the medial adjustment loop (Figure 5). Prior to final fixation, fluoroscopy with a C‐arm is used to confirm satisfactory joint reduction, and the position of the loop plate is re‐evaluated arthroscopically to ensure optimal placement.

**FIGURE 5:**
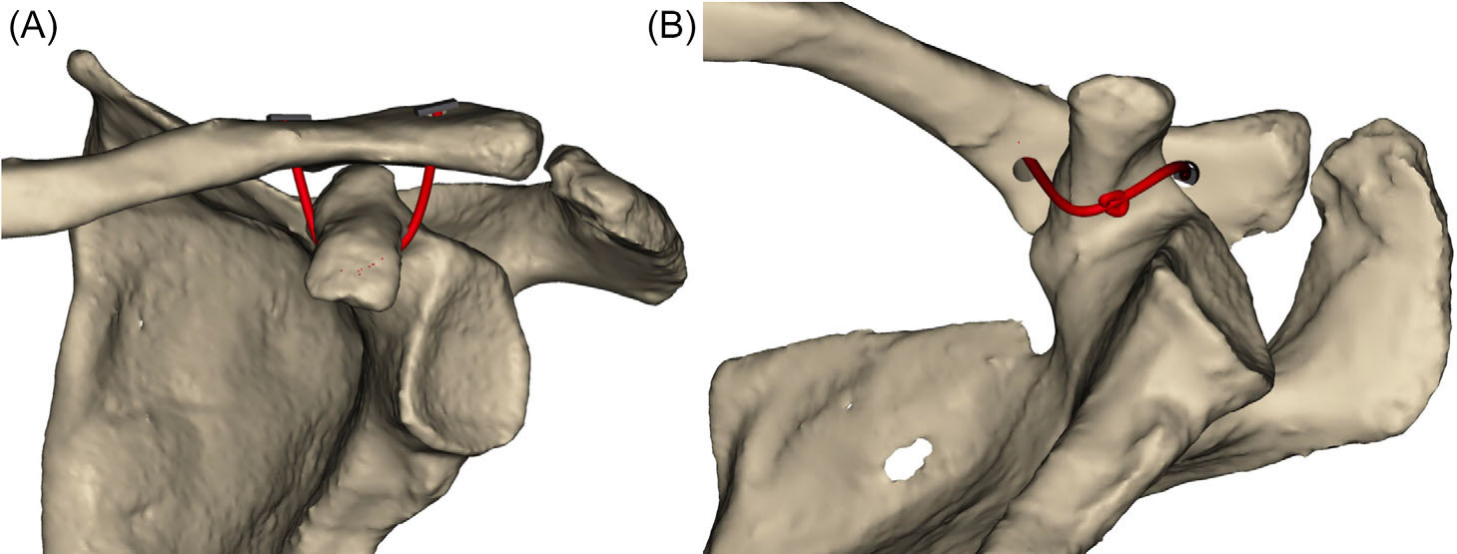
Schematic diagram illustrating the planned bone tunnel positions and Kirschner wire placement. (A) Planned bone tunnel positions before Kirschner wire insertion. (B) Kirschner wires inserted through the planned bone tunnels.

### Postoperative Rehabilitation Protocol

Radiographic evaluation is performed on postoperative day 1. Patients are instructed to wear a shoulder abduction brace postoperatively. Passive range‐of‐motion exercises are initiated on postoperative day 1 and performed daily. At 6 weeks postoperatively, full passive range‐of‐motion training is permitted. Progressive strengthening exercises are commenced at 2 months postoperatively, followed by supervised resistance training at 3 months. Participation in contact sports or high‐impact activities is prohibited for 6 months postoperatively.

### Ethics Approval and Consent to Participate

All procedures performed in studies involving human participants were in accordance with the ethical standards of the institutional and/or national research committee at the Quzhou People's Hospital and with the 1964 Helsinki Declaration and its later amendments or comparable ethical standards. Given the retrospective nature of this study, the informed consent was waived. Ethical approval for this study was obtained from Quzhou Affiliated Hospital of Wenzhou Medical University, Quzhou People's Hospital.

## DISCUSSION

This article describes an arthroscopy‐based double EndoButton adjustable‐loop plate fixation technique for the treatment of acute acromioclavicular joint dislocation. Through the design of a double EndoButton construct and an adjustable loop, this technique achieves anatomical reduction while providing continuous and stable mechanical support, aligning with the physiological loading characteristics of the acromioclavicular joint.

Compared with traditional hook plate fixation, this technique avoids the impingement of the implant on the subacromial space, thereby reducing the risks of postoperative subacromial impingement, rotator cuff injury, and the need for secondary surgery for implant removal. In contrast to single‐suspension systems (e.g., TightRope), the dual EndoButton construct creates a “double‐pulley”‐like mechanical effect, significantly enhancing fixation stability and rotational resistance, which is more conducive to controlling horizontal and vertical displacement of the distal clavicle.^11^ Furthermore, its adjustable‐loop design allows for real‐time, precise intraoperative adjustment of tension and coracoclavicular distance, facilitating not only anatomical reduction with sustained compression but also providing a more favorable mechanical environment for coracoclavicular ligament healing.

This dynamic fixation mechanism maintains biomechanical stability while permitting physiological micromotion of the clavicle, aligning with the functional demands of the shoulder joint. Biomechanical studies have shown that the failure load of this adjustable‐loop system can reach 1086 to 1365 N, which exceeds the ultimate load of the intact coracoclavicular ligament complex reported in cadaveric studies (approximately 500‐700 N).^12^ On the coracoid side, the system employs a stress‐dispersing design to reduce direct pressure on the bone surface, helping to mitigate the risks of bone erosion and stress fracture. Unlike traditional button‐suture systems that rely on manual knot tying, this technique uses a knotless self‐locking design, achieving stability through internal friction within the loop structure, thereby avoiding surgical failure because of knot loosening or failure.^13^, ^14^ Additionally, the knotless design reduces subcutaneous foreign body sensation and local irritation, contributing to improved postoperative patient comfort and satisfaction.

The arthroscopic approach offers minimally invasive advantages, enabling simultaneous exploration and management of concomitant injuries within the glenohumeral joint. It minimizes soft‐tissue dissection, reduces the risk of infection, and contributes to decreased postoperative pain and accelerated rehabilitation. This preliminary study confirms the safety of this technique for early postoperative functional rehabilitation, allowing patients to initiate guided joint mobilization early, which is beneficial for preventing joint stiffness and muscle atrophy.

However, this technique presents a learning curve, requiring surgeons to be familiar with the pericoracoid anatomy and instrument handling skills under arthroscopy. Long‐term follow‐up outcomes, large‐sample comparative studies with traditional surgical methods, and further biomechanical validation remain important directions for future investigation. In summary, arthroscopic double EndoButton adjustable‐loop plate fixation provides a minimally invasive solution for acute acromioclavicular joint dislocation that is characterized by minimal trauma, reliable fixation, and adherence to biomechanical principles, showing promising clinical application prospects. The pearls and pitfalls of this technique are summarized in Table 1, whereas its advantages and disadvantages are presented in Table 2. The surgical procedure is shown in Video 1.

**TABLE 1:** Pearls and Pitfalls

**Pearls**	**Pitfalls**
Preoperative 3D reconstruction using Mimics software helps accurately plan the positions of bilateral coracoid bone tunnels and measure the distance to the distal clavicle.	Failure to ensure that the drill bit penetrates only the bicortical clavicle can injure the subclavian vessels or brachial plexus.
Using 2 long needles to localize the anterior and posterior clavicular borders before drilling minimizes the risk of neurovascular injury.	Residual soft tissue around the coracoid process can obscure visualization and hinder suture passage.
Tightening the lateral adjustable loop first, followed by the medial loop, achieves more controlled and stable reduction.	Overtightening the loops may cause over‐reduction or joint stiffness; real‐time fluoroscopy is recommended during final tensioning.
The knotless, self‐locking design of the adjustable loop eliminates complications related to manual knot tying (e.g., loosening or failure).	Allowing uncontrolled active motion or resistance too early may lead to loss of reduction or implant failure.
Early passive range‐of‐motion exercise is permitted postoperatively because of the dynamic fixation mechanism.	

3D, 3‐dimensional.

**TABLE 2:** Advantages and Disadvantages

**Advantages**	**Disadvantages**
Minimally invasive arthroscopic approach reduces soft‐tissue dissection, blood loss, and postoperative pain.	The technique requires advanced arthroscopic skills and familiarity with pericoracoid anatomy, leading to a learning curve.
The double EndoButton construct provides a “double‐pulley” mechanical effect, enhancing rotational and translational stability than single‐suspension devices.	Intraoperative fluoroscopy is necessary to confirm reduction quality, which adds radiation exposure.
Adjustable‐loop design allows intraoperative fine‐tuning of tension and coracoclavicular distance, enabling precise anatomical reduction.	Long‐term outcomes and large‐sample comparative studies are still lacking.
Dynamic fixation permits physiological clavicular micromotion, potentially promoting ligament healing and reducing stress shielding.	Not suitable for chronic dislocations (>3 wk) or cases with severe comminuted fractures of the coracoid or clavicle.
No need for routine implant removal surgery because the low‐profile EndoButton plates rarely cause subacromial impingement or irritation.	

wk, weeks.

## DISCLOSURES

The authors (S.H., Z.Z., C.Q., H.Y., H.B.) declare that they have no known competing financial interests or personal relationships that could have appeared to influence the work reported in this article.
